# Selective Hydrolysis of Transferrin Promoted by Zr-Substituted Polyoxometalates

**DOI:** 10.3390/molecules25153472

**Published:** 2020-07-30

**Authors:** Laura S. Van Rompuy, Nada D. Savić, Alvaro Rodriguez, Tatjana N. Parac-Vogt

**Affiliations:** Department of Chemistry, KU Leuven, Celestijnenlaan 200F, 3001 Leuven, Belgium; laura-vr@hotmail.com (L.S.V.R.); nada.savic@kuleuven.be (N.D.S.); alrodi@gmail.com (A.R.)

**Keywords:** polyoxometalate, zirconium, metalloproteases, transferrin, hydrolysis

## Abstract

The hydrolysis of the iron-binding blood plasma glycoprotein transferrin (Tf) has been examined at pH = 7.4 in the presence of a series of Zr-substituted polyoxometalates (Zr-POMs) including Keggin (Et_2_NH_2_)_10_[Zr(PW_11_O_39_)_2_]∙7H_2_O (**Zr-K 1:2**), (Et_2_NH_2_)_8_[{*α*-PW_11_O_39_Zr-(*μ*-OH) (H_2_O)}_2_]∙7H_2_O (**Zr-K 2:2**), Wells-Dawson K_15_H[Zr(*α*_2_-P_2_W_17_O_61_)_2_]·25H_2_O (**Zr-WD 1:2**), Na_14_[Zr_4_(*α*-P_2_W_16_O_59_)_2_(*μ*_3_-O)_2_(*μ*-OH)_2_(H_2_O)_4_]·57H_2_O (**Zr-WD 4:2**) and Lindqvist (Me_4_N)_2_[ZrW_5_O_18_(H_2_O)_3_] (**Zr-L 1:1**), (nBu_4_N)_6_[(ZrW_5_O_18_(*μ*–OH))_2_]∙2H_2_O (**Zr-L 2:2**)) type POMs. Incubation of transferrin with Zr-POMs resulted in formation of 13 polypeptide fragments that were observed on sodium dodecyl sulfate poly(acrylamide) gel electrophoresis (SDS-PAGE), but the hydrolysis efficiency varied depending on the nature of Zr-POMs. Molecular interactions between Zr-POMs and transferrin were investigated by using a range of complementary techniques such as tryptophan fluorescence, circular dichroism (CD), ^31^P-NMR spectroscopy, in order to gain better understanding of different efficiency of investigated Zr-POMs. A tryptophan fluorescence quenching study revealed that the most reactive **Zr-WD** species show the strongest interaction toward transferrin. The CD results demonstrated that interaction of Zr-POMs and transferrin in buffer solution result in significant secondary structure changes. The speciation of Zr-POMs has been followed by ^31^P-NMR spectroscopy in the presence and absence of transferrin, providing insight into stability of the catalysts under reaction condition.

## 1. Introduction

Proteins play fundamental and crucial roles in nearly all biological processes such as catalysis, signaling transduction, DNA and RNA synthesis, transport and immune response [1]. The total number of different proteins on the Earth is estimated to be up to 10 million [2]. As the structure of protein determines its function, investigating a protein’s structure is one of the most challenging tasks in order to elucidate its biological role [3]. In the field of proteomics, which is defined as a large-scale study of protein structure and function, mass spectrometry (MS) is an indispensable tools used to analyze proteins. However, bearing in mind the high complexity and large size of these biomolecules, the main approaches for identification and characterization of proteins by MS require controlled hydrolysis of proteins into smaller fragments.

“Bottom-up” protein analysis refers to the characterization of proteins analyzing peptide fragments which are derived from proteolytic digestion of intact proteins [4]. This method employs different enzymes, among which trypsin is the most frequently used. The main advantages of trypsin include high efficiency and specificity at a low cost; however, the exclusive use of trypsin also has certain disadvantages [5]. Trypsin cleaves peptide bonds at the carboxyl side of arginine and lysine amino acids, which generates many peptide fragments that are less than six residues long. These short peptides often cannot be uniquely identified by MS, which leads to an incomplete coverage of the examined proteome [6]. Ideally, intact proteins can be directly analyzed without prior fragmentation by top-down method, in which proteins are not enzymatically digested into peptides, however MS analysis of intact proteins is still a very complex process. This led to the rise of middle-down proteomics which employs the same principles as bottom-up proteomics, however, here longer peptide fragments (approximately 10 kDa) are being generated than the typical tryptic peptides (0.5–3 kDa). By producing longer peptides for the analysis, sample complexity is reduced, and sequence coverage is improved [7]. Currently, a limiting factor for middle down proteomics is the small pool of proteases available which generate fragments with suitable lengths. The currently available enzymes also frequently have a very high cost compared to trypsin, which prevents their use on a wide scale [8]. Moreover, one of the main problems is their limited selectivity, as well as poor activity in very narrow temperature and pH range [9]. Chemical digestion of proteins could therefore provide an interesting alternative for proteases [5], but the most frequently used chemical reagents require harsh conditions, cleave the proteins with partially selectivity and low yields [10]. Moreover, most of them are toxic and have ability to modify side chain of the amino acids [11,12]. Therefore, there is need for more efficient and selective chemical agents which would overcome all this side effects of presently used chemical agents and preserved their structural properties.

In the last few years our research group has been developing a new class of artificial metalloproteases based on metal-oxo-based materials and on metal-substituted polyoxometalates, which are able to selectively hydrolyze proteins under mild experimental conditions while producing larger peptide fragments [9,13,14,15,16,17,18,19,20,21]. Polyoxometalates are a large class of inorganic metal-oxygen clusters which are composed of transition metals in their highest oxidation state. Depending on their structural, physical, and chemical properties POMs have different applications in various fields of material science, photochemistry, medicine, and catalysis [22,23,24]. The interactions of POMs with proteins have been studied by different approaches and provided insight on the possible role of POMs in biological systems and their potential use in medicine [24,25,26,27,28,29,30]. The Keggin, Wells-Dawson and Lindqvist type of POMs are most frequently investigated POMs, and their structure can be substituted with strongly Lewis acidic metals such as Zr(IV), Hf(IV) or Ce(IV). Different Zr-substituted POMs were shown to hydrolytically cleave peptide bonds in different proteins including human serum albumin (HSA) [14,31], horse heart myoglobin (HHM) [9] or cytochrome c (cyt c) [16], which vary in size, charge and structure. Interestingly, Zr-substituted POMs have shown high affinity towards hydrolysis of peptide bonds next to amino acids with carboxylate groups in their side chains such as Asp and Glu residues [9,14,31,32,33]. The interactions between the negatively charged POMs and proteins typically occurs at positively charged areas on the protein surface, however in some cases POMs can also interact with amino acids via covalent binding [24,34]. Since the interactions between POMs and proteins depend on different factors such as size, shape, charge, and hydrophilicity of the POM the challenge is to understand how these factors influence the specificity and selectivity of protein hydrolysis. Therefore in order to further advance the application of POMs as artificial proteases, in this work we evaluate hydrolysis of a complex glycoprotein such as transferrin with a series of Zr-substituted POMs which differ in size, charge and the number of imbedded Zr(IV) ions, and study their interaction with the protein with the range of complementary techniques.

## 2. Results and Discussion

### 2.1. Hydrolysis of Transferrin by Zr-Substituted POMs

Human serum transferrin is a glycoprotein consisting of 679 amino acids (AA) with an approximate molar mass of 80 kDa and two N-linked and one O-linked glycan chains. Its structure consists of two lobes, N-lobe (336 AA) and C-lobe (343 AA), which each contain globular domains that interact to form a deep hydrophilic metal-binding site at their interface [35]. Each lobe contains one iron-binding site, in which iron forms a distorted octahedron that is coordinated to Asp-63, Tyr-95, Tyr-188, His-249, and two carbonate oxygen atoms [35]. The main function of the protein is to transport iron in human blood [36]. Approximately 30% of Tf molecules are iron-loaded (holotransferrin), which leaves the unloaded protein (apotransferrin) free for transport of other metals [36].

**Figure 1:**
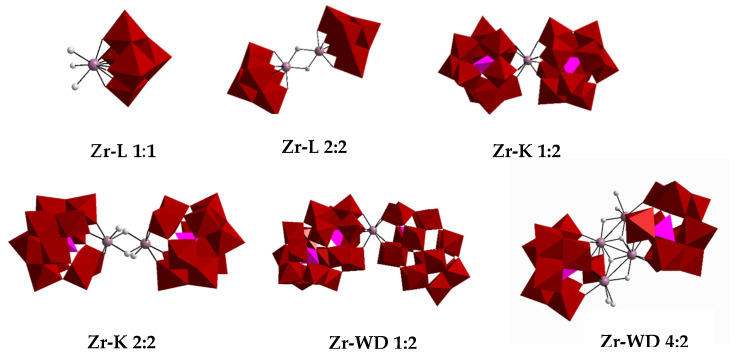
The polyhedral representation of Zr-substituted POMs (**Zr-L 1:1**, **Zr-L 2:2**, **Zr-K 1:2**, **Zr-K 2:2**, **Zr-WD 1:2** and **Zr-WD 4:2**). WO_6_ and PO_4_ are represented by red octahedrons and pink tetrahedrons, respectively. The grey spheres represent Zr(IV) ions.

A series of Zr(IV) POMs including (Et_2_NH_2_)_10_[Zr(PW_11_O_39_)_2_]·7H_2_O (**Zr-K 1:2**), (Et_2_NH_2_)_8_[{α-PW_11_O_39_Zr-(*μ*-OH)(H_2_O)}_2_]·7H_2_O (**Zr-K 2:2**), K_15_H[Zr(α_2_-P_2_W_17_O_61_)_2_]·25H_2_O (**Zr-WD 1:2**), Na_14_[Zr_4_(α-P_2_W_16_O_59_)_2_(*μ_3_*-O)_2_(*μ*-OH)_2_(H_2_O)_4_]∙57H_2_O (**Zr-WD 4:2**). (Me_4_N)_2_[ZrW_5_O_18_(H_2_O)_3_] (**Zr-L 1:1**), (nBu_4_N)_6_[(ZrW_5_O_18_(*μ*–OH))_2_]∙2H_2_O (**Zr-L 2:2**), (Figure 1) have been evaluated for their potential to hydrolyze transferrin. Solutions of transferrin (7.5 µM) was incubated with each Zr-substituted POM (7.5 mM) in phosphate buffer (10 mM, pH 7.4) at 60 °C over the course of a week. Although previous studies have shown that phosphate buffer may impact POM reactivity [37], it ensures pH stability and pH conditions which are pertinent to physiological conditions.

**Figure 2:**
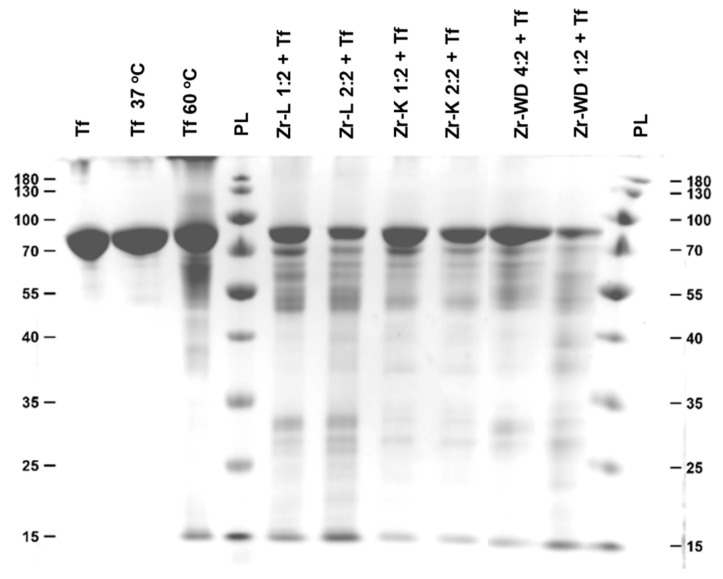
SDS-Page gel featuring intact transferrin (lane 1), transferrin incubated in phosphate buffer (lanes 2–3), and transferrin incubated in buffer solutions containing different metal-substituted POMs (lanes 5–10). The samples in this gel were incubated for 3 days.

Aliquots of the reaction mixture were taken at different time intervals and analyzed by sodium dodecyl sulfate–polyacrylamide gel electrophoresis (SDS-PAGE) on separate gels for each Zr-POM by plotting the different time increments next to each other (Figures S1–S6). The selectivity of different POMs was studied by analyzing the band pattern and the Mw of the bands on each gel. For ease of comparison, an additional gel was run which contained the aliquots taken after 3 days of hydrolysis for each POM (Figure 2). All POMs show hydrolysis after 24 h of incubation and the hydrolysis yield increases with time, with 13 polypeptide fragments being visible on the SDS-PAGE gel. Interestingly, all bands appeared at the same position in SDS-PAGE gel, indicating that hydrolysis occurred at the same region of the protein. Hydrolysis of transferrin in the presence of Ce-K 1:2 produced only nine polypeptide fragments on the SDS-PAGE, suggesting that the nature of the Lewis acid metal can affect the outcome of hydrolytic reaction [38]. The hydrolysis efficiencies of each Zr-POM were estimated by determining the intensities of the bands, which are proportional to the concentration of the polypeptide fragment that they represent. The intensity decreases of the band corresponding to the intact protein was used as a measure to estimate the efficiency of the hydrolysis for each POM. By comparing the intensities of the transferrin band after 3 and 7 days of hydrolysis with the band of the sample before incubation, hydrolysis efficiencies were obtained and were represented as the percentage of hydrolyzed protein (Table 1).

**Figure 3:**
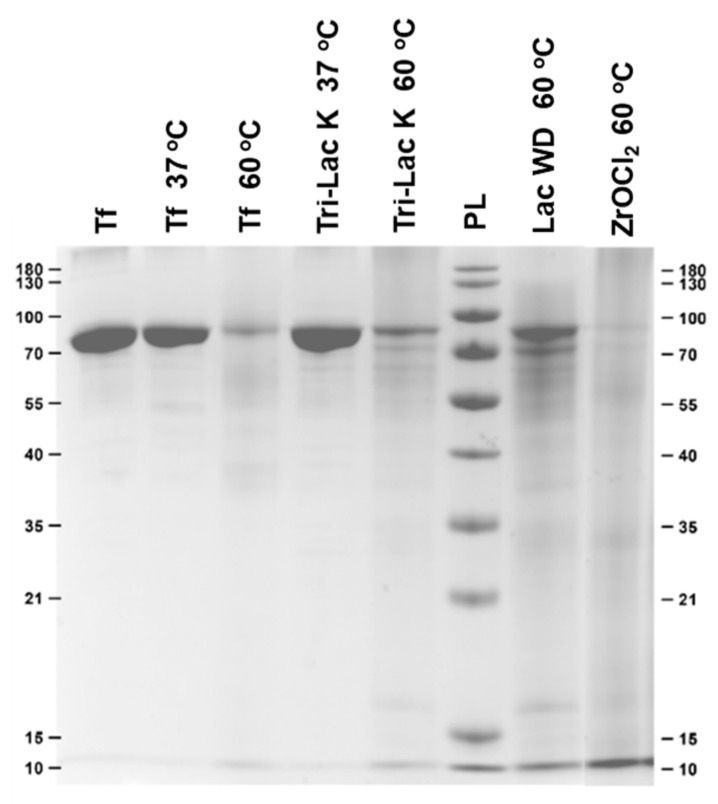
SDS-Page gel featuring intact transferrin (lane 1), transferrin incubated in phosphate buffer (lanes 2–3), and transferrin incubated in buffer solutions containing control samples. The samples were incubated for 7 days.

Several control reactions were performed in order to prove that the Zr-POMs are responsible for the observed hydrolytic reaction. Transferrin (7.5 µM) was incubated at different temperatures (37 and 60 °C) in the absence of Zr-POMs (Figure 3), as well as in the presence of the lacunary POMs (the POM scaffolds without the Zr(IV) ion) and with Zr salt (ZrOCl_2_) which was used for the synthesis of Zr-POMs (0.75 mM), in the phosphate buffer (10 mM, pH 7.4) up to 7 days (Figure 3). A limited and non-specific hydrolysis was observed in most of the control samples, which is most likely due to the prolonged incubation at elevated temperatures.

In the presence of ZrOCl_2_ a heavily smeared lane was detected without defined protein fragments, which made quantification of the control reaction difficult. This is in accordance with the previous reports using Zr(IV) salts, and could be explained by the formation of insoluble gels at neutral pH [14]. The controlled hydrolysis of transferrin with Zr-substituted POMs therefore relies on the combination of the POM scaffold and the hydrolytically active metal ion, where POM ligand stabilize Zr(IV) ion.

### 2.2. Interaction Between Transferrin and Zr-Substituted POMs

#### 2.2.1. Tryptophan Fluorescence Quenching Studies

**Figure 4:**
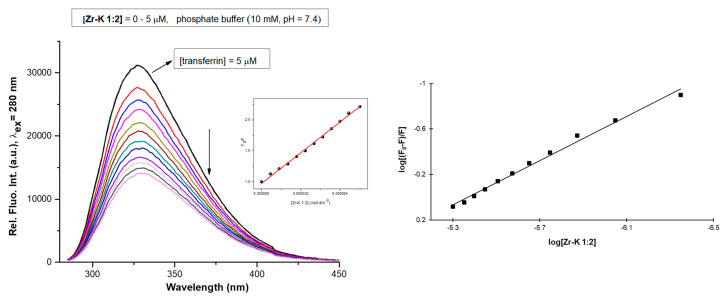
Tryptophan fluorescence quenching spectra of transferrin (5 µM) in phosphate buffer (10 mM, pH 7.4) with increasing concentrations of **Zr-K 1:2** (0–5 µM). The insert shows a plot of F_0_/F versus the POM concentration. (Right) Derived Stern-Volmer plot used to calculate the association constant.

Human transferrin possesses eight Trp residues [39], which are situated on the surface of the protein. Tryptophan fluorescence quenching experiments were performed in order to determine if the Zr-POMs interact with transferrin and whether the correlation exists between the interaction strength and the hydrolysis efficiency. Fluorescence spectra were recorded for transferrin (5 µM) in phosphate buffer (10 mM, pH 7.4), while the concentration of Zr-POMs was increased in stepwise from 0–5 µM. In this study, nearly linear relationship between F_0_/F and the Zr-POMs concentration was observed which indicate the static quenching of Trp fluorescence and formation of ground state complex (Figure 4 and Figures S7–S11). The same observation was noticed in other studies involving the interactions between metal substituted POMs and proteins [31,40,41]. In order to compare the affinities of all investigated Zr-POMs, association constants were calculated by fitting a derived Stern-Volmer equation (Equation (1)):

(1) $$\log(\frac{\left(F_{0}-F\right)}{F})\;{=\mathrm{logK}}_{a}+n\;\log\left[Q\right]$$

In Equation (1), (K_a_) represents the association constant, (*n*) is the number of quencher molecules and ([Q]) is the concentration of the quencher, while the measured fluorescence in each step is given by (*F*), and the fluorescence in absence of quencher by (*F*_0_). Derived Stern-Volmer equations were fitted for all of the tested Zr-POMs (Figure 4 and Figures S7–S11), and the obtained values are summarized in Table 2. In all cases decrease of Trp intensity was observed upon addition of Zr-POMs. Although possible POM equilibria and orientation of the POM can cause variation of the K_a_ [9], the overall results suggest that all investigated Zr-POMs interact with transferrin and that the strength of the interaction differs depending on the structure, size and charge of the Zr-POMs. Moreover, the percentage of hypochromic effect is in agreement with association constant (K_a_) (Table 2), since the highest association constant resulted in the most pronounced hypochromic effect.

For the Zr-L POMs, the 1:1 structure exhibits a stronger interaction than the 2:2 structure, suggesting that the smaller structure of the **Zr-L 1:1** can better approach the Trp residues on the protein surface. For Zr-K species, stronger interaction was seen for the 2:2 species compared to 1:2 Zr-K POM. However, it is well known that dimeric Zr-K structures undergo interconversion in solution, and can dissociate into their monomeric structure. This conversion is more favorable for **Zr-K 2:2** [14], resulting in formation of the smaller 1:1 Zr-K monomer [9,42] which can better fit on the transferrin surface, causing more efficient quenching of Trp fluorescence.

Interestingly, **Zr-WD 1:2** has shown the highest binding ability towards transferrin, which can be explained by the existence of equilibria between dimeric 1:2 and monomeric 1:1 species in which Zr(IV) has more available coordination sites to interact with the protein [14,17]. This type of equilibria has not been observed for the more rigid **Zr-WD 4:2** structure. Moreover, the net negative charges of **Zr-WD 1:2** (−16) and Zr-WD 1:1 (−6) are higher in comparison to the net negative charge of **Zr-WD 4:2** (−4) which can be one of the reasons for its better quenching efficiency.

Overall, the lower net negative charge of Zr-K 1:1 (−3), **Zr-L 1:1** (−2) and **Zr-L 2:2** (−4) results in weaker interaction between POM and positive patches of protein. In addition to electrostatic interactions, Zr(IV) ion can also interact with the protein residues, and therefore the higher number of Zr(IV) ions present in **Zr-WD 4:2** structure can also explain its better quenching affinity.

#### 2.2.2. ^31^P-NMR Stability Study

**Figure 5:**
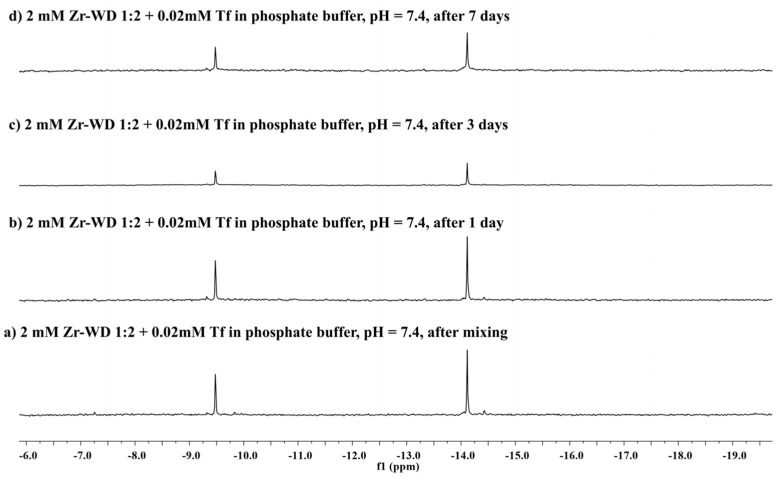
^31^P-NMR of **Zr-WD 1:2** (2 mM) incubated in the presence of transferrin (20 µM) in phosphate buffer (10 mM, pH 7.4) at 60 °C. Incubation times are shown in the figure. The **Zr-WD 1:2** structure (−14.11 and −9.548 ppm) remains largely stable over time.

In order to gain insight into the stability and speciation of Zr-substituted POMs in the presence of transferrin ^31^P-NMR spectroscopy was applied in the following step. Unfortunately, the POM speciation using ^31^P-NMR cannot be determined under the same conditions that were used for Trp fluorescence quenching studies ^31^P-NMR requires much higher concentrations of POMs in order for them to be detected. Therefore, modified reaction conditions were used to determine speciation behavior of Zr-substituted POMs and whether the presence of transferrin influences this behavior. Zr-L structure was not investigated since this POM does not contain phosphorus atom. Solutions containing Zr-POMs (2 mM) were prepared in 10 mM phosphate buffer at pH 7.4 in presence of transferrin (20 μM) and in the absence of transferrin in buffer solution and in water. All solutions of investigated Zr-POMs in the presence of protein were incubated at 60 °C up to 7 days. ^31^P-NMR spectra were measured immediately after mixing and after 1, 3, and 7 days (Figure 5 and Figures S12–S22), and were referenced to 25% H_3_PO_4_ which was used as an external standard.

Figure 5 indicates that the stability of **Zr-WD 1:2** in the absence and presence of transferrin remains unaffected since no additional signals were observed in the spectra after 7 days. Similarly to dimeric Zr-K POMs, **Zr-WD 1:2** is also known to undergo equilibria between dimeric and monomeric species in the solution, but this process is too fast on the NMR scale for the monomeric species to be detected in ^31^P-NMR spectra [16]. However, the existence of 1:1 Zr-WD and 1:1 Zr-K monomeric species was proven by single crystal X-ray diffraction of a non-covalent complexes that were formed with HEWL protein [43,44]. Interestingly, such equilibria were not observed for the more rigid **Zr-WD 4:2,** which can be an explanation for its lower activity. In analogy to **Zr-WD 1:2,** the rapid dissociation of **Zr-K 1:2** into monomeric species was not observed in ^31^P-NMR spectra under applied experimental conditions (pH = 7.4). Appearance of an additional signal at −10.5 ppm indicates that lacunary Keggin is also present in solution, and its formation can be attributed to the presence of the phosphate buffer. The lacunary Keggin POM was observed immediately upon dissolution of **Zr-K 1:2** in the phosphate buffer, regardless of the protein presence (Figures S12,S14), while in the pure D_2_O the formation of lacunary Keggin was not be observed (Figure S13). The presence of the lacunary Keggin in **Zr-K 1:2** solution can also explain the lower hydrolytic activity of this POM towards transferrin. Interestingly, **Zr-K 2:2** was almost fully converted to **Zr-K 1:2** in buffer solution both in the presence and in absence of transferrin (Figures S15 and S17), while in pure D_2_O solutions the **Zr-K 2:2** remained stable (Figure S16).

#### 2.2.3. Circular Dichroism (CD) Spectroscopy

**Figure 6:**
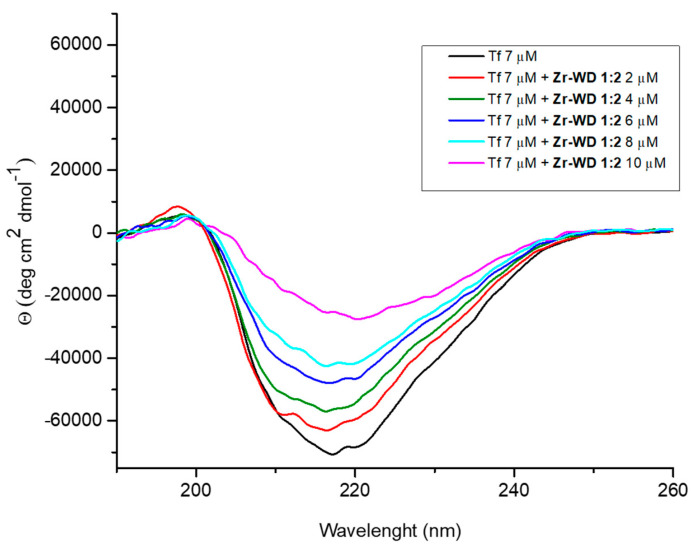
CD spectra of transferrin (7 µM) in phosphate buffer (10 mM, pH 7.4) with increasing concentrations (0–10 µM) of **Zr-WD 1:2**.

The interactions between Zr-POMs and transferrin were further investigated by CD spectroscopy. Circular dichroism is a convenient technique for monitoring the changes in the secondary structure of a protein upon interaction with external ligands. Circular dichroism spectra of transferrin (7 µM) were measured in phosphate buffer (10 mM, pH 7.4) in the absence and in the presence of Zr-POMs (0–10 µM) at room temperature immediately after mixing (Figure 6 and Figures S23–S27). The UV-CD spectra of free protein has intense negative band around 220 nm, which is characteristic of a protein having secondary structure that consists of alternating α helices and β sheets [45]. Addition of all investigated Zr-POMs to transferrin resulted in the weakening of the negative ellipticity, which is indication of the binding and the formation of a more loose protein conformation.

However, some differences in the CD spectra could be observed depending on the nature of Zr-POM. Compared to smaller Lindqvist and Keggin POMs, the larger Wells-Dowson structures caused more pronounced changes in secondary structure even when added in low concentrations. Similar results were reported previously in the studies involving Zr-POMs and cyt c [16]. The red shift observed in the spectra after addition of **Zr-WD 1:2** (Figure 6), indicates that the interaction between transferrin and polyoxometalate slightly changed the micro-environment of the protein [46]. A similar decrease in the CD signal intensity which was accompanied by a red shift was also observed for **Zr-WD 4:2** POM, suggesting that more pronounced structural changes were required in the secondary structure of protein in order to accommodate larger Zr-WD species [46].

## 3. Materials and Methods

### 3.1. Materials

Phosphotungstic acid, sodium tungstate dihydrate, coomassie brilliant blue G-250, sodium dodecyl sulfate, sodium bicarbonate, sodium carbonate, were purchased from Acros Organics (Fair Lawn, NJ, USA). Transferrin, glycine, ammonium persulfate, tetramethylammonium chloride, tetrabutylammonium hydroxide, zirconyl chloride octahydrate, zirconium(IV) chloride, sodium phosphate dibasic and deuterium oxide were bought from Sigma-Aldrich (St. Louis, MO, USA). Acetone, sodium phosphate monobasic, diethyl ether, methanol, isopropanol, ammonium chloride, sodium chloride, sodium sulfate, sodium perchlorate monohydrate were obtained from VWR Chemicals (Radnor, PA, USA). Phosphoric acid (85%), potassium bicarbonate, hydrochloric acid, sulfuric acid, ethanol (absolute) were purchased from Fisher Chemicals (Hampton, NH, USA). Acrylamide (30%), tris(hydroxymethyl)aminomethane and potassium chloride were bought from AppliChem Panreac (Darmstadt, Germany). Acetic acid glacial was obtained from CHEM-LAB (Zedelgem, Belgium). 2-mercaptoethanol was purchased from Merck (Darmstadt, Germany). Trichloroacetic acid was bought from Janssen Chimica (Beerse, Belgium). Diethylamine hydrochloride was obtained from Alfa Aesar (Haverhill, MA, USA). PageRuler Prestained Protein Ladder was purchased from Thermo Scientific (Waltham, MA, USA)). Bromophenol blue was bought from Fluka Analytical (Buchs, Switzerland).

### 3.2. Methods

(Et_2_NH_2_)_10_[Zr(PW_11_O_39_)_2_]·7H_2_O [47], (Et_2_NH_2_)_8_[{*α*-PW_11_O_39_Zr-(*μ*-OH)(H_2_O)}_2_]·7H_2_O [47], K_6_[P_2_W_18_O_62_]·14/19H_2_O [48], K_10_[*α_2_*-P_2_W_17_O_61_]∙20H_2_O [48], K_15_H[Zr(*α*_2_-P_2_W_17_O_61_)_2_]·25H_2_O [47], Na_9_[A-*α*-PW_9_O_34_]∙16H_2_O [48], (Me_4_N)_2_[ZrW_5_O_18_(H_2_O)_3_] [49], (*n-*Bu_4_N)_6_[(ZrW_5_O_18_(*μ*–OH))_2_]∙2H_2_O [49] and Na_14_[Zr_4_(*α*-P_2_W_16_O_59_)_2_(*μ*_3_-O)_2_(*μ*-OH)_2_(H_2_O)_4_]∙57H_2_O [50] were synthesized according to published procedures.

#### 3.2.1. Hydrolisis Experiments

Solutions containing 7.5 µM of transferrin and 7.5 mM of investigated POMs were prepared in sodium phosphate buffer solution at pH 7.4. Samples were incubated in a Thermomixer (Eppendorf, Hamburg, Germany) in microcentrifuge tubes at 60 °C for 7 days. Aliquots were taken in different time intervals. SDS-PAGE analysis was performed on a 12% Laemmli gels,[51] which were stained with a colloidal Coomassie solution [52]. Each sample (15 µL) was mixes with sample buffer (5 µL) and heated 95 °C for 5 min. 10 µL of the resulting solution was loaded to the gel. A page ruler prestained protein ladder (10–170 kDa) was used as a standard. Two SDS page gels are run at the same time in the Tris-glycine-SDS running buffer with voltage of 200 V, constant current set to 70 mA and maximum power set to 50 W. Total running time was approximately 1.5 h. The image of each gel was analyzed with Image Lab software (Bio-Rad, Hercules, CA, USA).

#### 3.2.2. Fluorescence Spectroscopy

Tryptophan fluorescence quenching studies of buffered 5 μM transferrin solutions (10 mM sodium phosphate buffer, pH = 7.4) at room temperature were recorded from 285 to 450 nm, with a maximum at approximately 330 nm. Excitation of the sample took place at 280 nm. Fluorescence spectra were measured with increasing concentrations of Zr-POMs. The stock solutions of Zr-POMs were prepared 1 day before experiments were done. Three µL aliquots of 450 µM Zr-POM solutions were added to the protein solution until a final POM concentration of 5 µM was reached. Measurements were performed in triplicate 5 min after each addition. Spectra were recorded on a FS-920P spectrofluorimeter (Edinburgh Instruments, Livingston, UK). Data were analysed in Origin Pro 8.0 (OriginLab Corporation, Northampton, MA, USA).

#### 3.2.3. Circular Dichroism Spectroscopy

Solutions containing 7 μM transferrin and 0 to 10 μM of investigated POMs were prepared in 10 mM phosphate buffer solution, pH = 7.4. The stock solutions of Zr-POMs were prepared 1 day before experiments were done. CD spectra were measured immediately after mixing the protein with certain amount of Zr-POMs. Quartz cuvettes with an optical path length of 1 mm were used. CD spectra were recorded using a J-1500 Circular Dichroism spectrometer (Jasco, Oklahoma City, OK, USA). The buffer solution was measured for baseline correction. Origin Pro 8.0 was used for data analysis.

#### 3.2.4. ^31^P-NMR Spectroscopy

All ^31^P-NMR spectra were recorded on an Avance 400 (161.98 MHz) spectrometer (Bruker, Billerica, MA, USA). Solutions containing 2 mM of investigated POMs and 20 μM transferrin were prepared in 10 mM pH 7.4 phosphate buffer. Moreover, solutions containing investigated POMs without protein were prepared in 10 mM pH 7.4 phosphate buffer and D_2_O. ^31^P-NMR spectra of all samples were measured at different incubation times up to 7 days. For each measurement, 600 μL of the sample was taken and 2 drops of D_2_O were added. As an external standard, 25% H_3_PO_4_ in D_2_O in a sealed capillary was used.

## 4. Conclusions

This study has shown that transferrin, a relatively large glycoprotein, was selectively hydrolyzed by a series of Zr-POMs under physiological pH conditions. The observed cleavage patterns were the same for all of the investigated Zr-POMs, however the efficiency of hydrolysis decreased in the following order: **Zr-WD 1:2** > **Zr-WD 4:2** > **Zr-K 2:2** > **Zr-L 2:2** > **Zr-L 1:1** > **Zr-K 1:2**. The results of Trp-fluorescence quenching studies have shown that Zr-WD forms have the highest association constant, which is in accordance with their high hydrolytic activity. Based on the net negative charge of examined Zr-POMs, it is plausible that the electrostatic binding between Zr-POMs and the positive regions of the transferrin which are accessible for the interaction, are the main driving force for the interaction. The equilibria which exist between dimeric **Zr-WD 1:2** and monomeric Zr-WD 1:1 species are likely to facilitate the reactivity, since Zr-WD 1:1 is presumed to be the hydrolytically active species. ^31^P-NMR studies have shown that Zr-K POMs are less stable in buffer solution in the presence of transferrin, which might explain their lower hydrolytic activity. Addition of increasing concentrations of Zr-POMs resulted in the large changes of the secondary structure of transferrin, which were most pronounced for the large Zr-WD POMs. These results indicate that partial unfolding of transferrin facilitates accommodation of Zr-POMs on the protein surface, and that the type of POM scaffold and the net negative charge play a key role in the binding efficiency.

## Figures and Tables

**Table 1:** Hydrolysis efficiencies of investigated POMs toward transferrin after 3 and 7 days of incubation.

POM	Hydrolysis Efficiencies (%)	
	After 3 Days	After 7 Days
**Zr-WD 1:2**	79	97
**Zr-WD 4:2**	64	92
Zr-K 2:2 *	45	82
Zr-K 1:2 *	48	73
**Zr-L 1:1**	48	80
**Zr-L 2:2**	50	81
**Tri-Lac K 37 °C**	/	0
**Tri-Lac K 60 °C**	/	~4
**Lac WD 60 °C**	/	~6

* Dimeric Zr-K forms (**Zr-K 1:2** and **Zr-K 2:2**) are pre-catalysts which are in equilibrium with monomeric (Zr-K 1:1) form. /: hydrolysis efficiency was not calculated.

**Table 2:** Calculated values of association constant (K_a_), the number of bound molecules (*n*), the percentage of hypochromic effect and percentage of hydrolyzed protein.

POM	Hypochromic Effect (%)	*n*	Ka (M^−1^)	R^2^	Hydrolysis (%) After 7 Days
**Zr-WD 1:2**	83.04	1.52	5.71 × 10^8^	0.9920	97
**Zr-WD 4:2**	80.92	1.30	3.02 × 10^7^	0.9805	92
Zr-K 2:2 *	66.92	1.13	2.17 × 10^6^	0.9878	82
Zr-K 1:2 *	53.75	0.97	1.67 × 10^5^	0.9921	73
**Zr-L 1:1**	36.60	0.97	8.22 × 10^4^	0.9975	80
**Zr-L 2:2**	30.84	0.85	1.61 × 10^4^	0.9721	81

* Dimeric Zr-K forms (**Zr-K 1:2** and **Zr-K 2:2**) are pre-catalysts which are in equilibrium with monomeric (Zr-K 1:1) form.

## Supplementary Materials

The following are available online: (Figures S1–S27; SDS-PAGE gels, Tryptophan fluorescence quenching spectra, Stern-Volmer plots, ^31^P-NMR spectra, CD spectra).
