# Supplementary material for: Selective Hydrolysis of Transferrin Promoted by Zr-Substituted Polyoxometalates
Source: Molecules. 2020 Jul 30;25(15):3472. doi: 10.3390/molecules25153472 (PMC7435656; doi:10.3390/molecules25153472)
Supplement: Supplementary file 1 [file molecules-25-03472-s001.pdf]

Supplementary Material

Selective Hydrolysis of Transferrin Promoted by Zr-Substituted Polyoxometalates

Laura S. Van Rompuy, Nada D. Savić, Alvaro Rodriguez and Tatjana N. Parac-Vogt *

Department of Chemistry, KU Leuven, Celestijnenlaan 200F, 3001 Leuven, Belgium
* Correspondence: tatjana.vogt@kuleuven.be; Tel.: +32-16-32-76-12

TABLE OF CONTENTS

Description	Page
**Figure S1.** SDS-PAGE gel of transferrin (7.5 μM) in water (lane 1), transferrin (7.5 μM) incubated in phosphate buffer (10 mM, pH 7.4) for 7 days at 60 °C (lane 2) and transferrin (7.5 μM) incubated in phosphate buffer (10 mM, pH 7.4) with **Zr-WD 1:2** (0.75 mM) at 60 °C for different time increments (lanes 4-9).	**S6**
**Figure S2.** SDS-PAGE gel of transferrin (7.5 μM) in water (lane 1) and transferrin (7.5 μM) incubated in phosphate buffer (10 mM, pH 7.4) with **Zr-WD 4:2** (0.75 mM) at 60 °C for different time increments (lanes 3-9).	**S6**
**Figure S3.** SDS-PAGE gel of transferrin (7.5 μM) in water (lane 1), transferrin (7.5 μM) incubated in phosphate buffer (10 mM, pH 7.4) for 7 days at 60 °C (lane 2) and transferrin (7.5 μM) incubated in phosphate buffer (10 mM, pH 7.4) with **Zr-K 1:2** (0.75 mM) at 60 °C for different time increments (lanes 4-9).	**S7**
**Figure S4.** SDS-PAGE gel of transferrin (7.5 μM) in water (lane 1), transferrin (7.5 μM) incubated in phosphate buffer (10 mM, pH 7.4) for 7 days at 60 °C (lane 2) and transferrin (7.5 μM) incubated in phosphate buffer (10 mM, pH 7.4) with **Zr-K 2:2** (0.75 mM) at 60 °C for different time increments (lanes 4-9).	**S7**
**Figure S5.** SDS-PAGE gel of transferrin (7.5 μM) in water (lane 1), transferrin (7.5 μM) incubated in phosphate buffer (10 mM, pH 7.4) for 7 days at 60 °C (lane 2) and transferrin (7.5 μM) incubated in phosphate buffer (10 mM, pH 7.4) with **Zr-L 1:1** (0.75 mM) at 60 °C for different time increments (lanes 4-9).	**S8**
**Figure S6.** SDS-PAGE gel of transferrin (7.5 μM) in water (lane 1) and transferrin (7.5 μM) incubated in phosphate buffer (10 mM, pH 7.4) with **Zr-L 2:2** (0.75 mM) at 60 °C for different time increments (lanes 3-9).	**S8**
**Figure S7.** Tryptophan fluorescence quenching spectra of transferrin (5 μM) in phosphate buffer (10 mM, pH 7.4) with increasing concentrations of **Zr-WD 1:2** (0-5 μM). The insert shows a plot of F_0/F versus the POM concentration. (Right) Derived Stern-Volmer plot used to calculate the association constant.	**S9**

**Figure S8.** Tryptophan fluorescence quenching spectra of transferrin (5 μM) in phosphate buffer (10 mM, pH 7.4) with increasing concentrations of **Zr-WD 4:2** (0-5 μM). The insert shows a plot of F_0/F versus the POM concentration. (Right) Derived Stern-Volmer plot used to calculate the association constant.	**S9**
**Figure S9.** Tryptophan fluorescence quenching spectra of transferrin (5 μM) in phosphate buffer (10 mM, pH 7.4) with increasing concentrations of **Zr-K 2:2** (0-5 μM). The insert shows a plot of F_0/F versus the POM concentration. (Right) Derived Stern-Volmer plot used to calculate the association constant.	**S10**
**Figure S10.** Tryptophan fluorescence quenching spectra of transferrin (5 μM) in phosphate buffer (10 mM, pH 7.4) with increasing concentrations of **Zr-L 1:1** (0-5 μM). The insert shows a plot of F_0/F versus the POM concentration. (Right) Derived Stern-Volmer plot used to calculate the association constant.	**S10**
**Figure S11.** Tryptophan fluorescence quenching spectra of transferrin (5 μM) in phosphate buffer (10 mM, pH 7.4) with increasing concentrations of **Zr-L 2:2** (0-5 μM). The insert shows a plot of F_0/F versus the POM concentration. (Right) Derived Stern-Volmer plot used to calculate the association constant.	**S11**
**Figure S12.** ^{31}P NMR of **Zr-K 1:2** (2 mM) in phosphate buffer (10 mM, pH 7.4) at room temperature. The **Zr-K 1:2** structure (-14.78 and -14.83 ppm) remains largely stable over time, the signal at -10.85 ppm is attributed to the lacunary Keggin.	**S12**
**Figure S13.** ^{31}P NMR of **Zr-K 1:2** (2 mM) in D_2O at room temperature. The **Zr-K 1:2** structure (-14.78 and -14.83 ppm) remains largely stable over time.	**S12**
**Figure S14.** ^{31}P NMR of **Zr-K 1:2** (2 mM) in the presence of transferrin (20 μM) incubated in phosphate buffer (10 mM, pH 7.4) at 60 °C. The **Zr-K 1:2** structure (-14.78 and -14.83 ppm) remains largely stable over time, the signal at -10.85 ppm is attributed to the lacunary Keggin.	**S13**
**Figure S15.** ^{31}P NMR of **Zr-K 2:2** (2 mM) in phosphate buffer (10 mM, pH 7.4) at room temperature. The peak corresponding to intact **Zr-K 2:2** appears at -13.69 ppm in buffer solution. Simultaneously, 2 peaks corresponding to **Zr-K 1:2** appeared around -14.77 and 14.86 ppm. Additional peaks appeared around -14 ppm and one around -3 ppm.	**S14**

**Figure S16.** ^{31}P NMR of **Zr-K 2:2** (2 mM) incubated in D_2O at room temperature. The **Zr-K 2:2** structure (-14.78 and -14.83 ppm) remains largely stable over time.	**S15**
**Figure S17.** ^{31}P NMR of **Zr-K 2:2** (2 mM) incubated in phosphate buffer (10 mM, pH 7.4) at 60 °C in the presence of transferrin (20 μM). The peak corresponding to intact **Zr-K 2:2** appears at -13.69 ppm in buffer solution, this peak disappeared after 1 day of incubation. Simultaneously, 2 peaks corresponding to **Zr-K 1:2** appeared around -14.77 and 14.86 ppm. Additional peaks appeared around -14 ppm and one around -3 ppm immediately after mixing. In all conditions, **Zr-K 2:2** was quickly converted to **Zr-K 1-2** upon incubation. Other unknown peaks appeared around -11 ppm.	**S16**
**Figure S18.** ^{31}P NMR of **Zr-WD 1:2** (2 mM) in phosphate buffer (10 mM, pH 7.4) at room temperature. The **Zr-WD 1:2** structure (-14.11 and -9.48 ppm) remains largely stable over time.	**S17**
**Figure S19.** ^{31}P NMR of **Zr-WD 1:2** (2 mM) in D_2O at room temperature. The **Zr-WD 1:2** structure (-14.08 and -9.47 ppm) remains largely stable over time.	**S18**
**Figure S20**. ^{31}P NMR of **Zr-WD 4:2** (2 mM) in phosphate buffer (10 mM, pH 7.4) at room temperature. The **Zr-WD 4:2** structure (-14.59 and -7.28 ppm) remains largely stable over time.	**S19**
**Figure S21.** ^{31}P NMR of **Zr-WD 4:2** (2 mM) in D_2O at room temperature. The **Zr-WD 4:2** structure (-14.579 and -7.28 ppm) remains largely stable over time.	**S20**
**Figure S22.** ^{31}P NMR of **Zr-WD 4:2** (2 mM) incubated in phosphate buffer (10 mM, pH 7.4) at 60 °C in the presence of transferrin (20 μM). The **Zr-WD 4:2** structure (-14.59 and -7.28 ppm) remains largely stable over time.	**S21**
**Figure S23.** CD spectra of transferrin (7 μM) in phosphate buffer (10 mM, pH 7.4) with increasing concentrations (0–10 μM) of **Zr-WD 4:2**.	**S22**
**Figure S24.** CD spectra of transferrin (7 μM) in phosphate buffer (10 mM, pH 7.4) with increasing concentrations (0–10 μM) of **Zr-K 1:2**.	**S22**
**Figure S25.** CD spectra of transferrin (7 μM) in phosphate buffer (10 mM, pH 7.4) with increasing concentrations (0–10 μM) of **Zr-K 2:2**.	**S23**
**Figure S26.** CD spectra of transferrin (7 μM) in phosphate buffer (10 mM, pH 7.4) with increasing concentrations (0–10 μM) of **Zr-L 1:1.**	**S23**

**Figure S27.** CD spectra of transferrin (7 μM) in phosphate buffer (10 mM, pH 7.4) with increasing concentrations (0–10 μM) of **Zr-L 2:2**.	**S24**

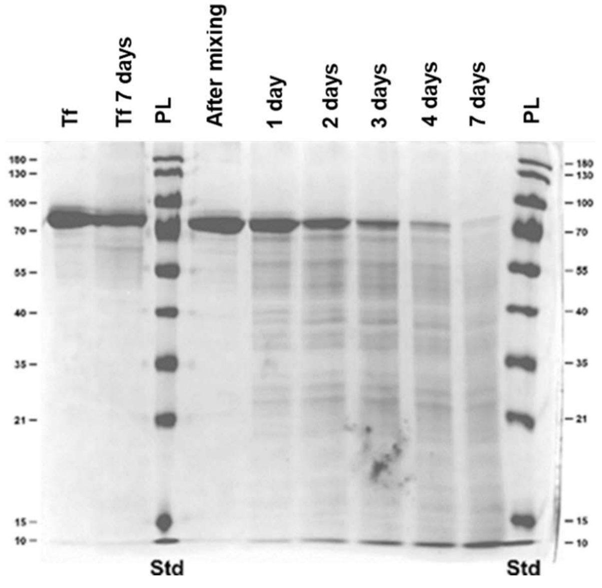

Figure S1. SDS-PAGE gel of transferrin (7.5 μM) in water (lane 1), transferrin (7.5 μM) incubated in phosphate buffer (10 mM, pH 7.4) for 7 days at 60 °C (lane 2) and transferrin (7.5 μM) incubated in phosphate buffer (10 mM, pH 7.4) with **Zr-WD 1:2** (0.75 mM) at 60 °C for different time increments (lanes 4–9).

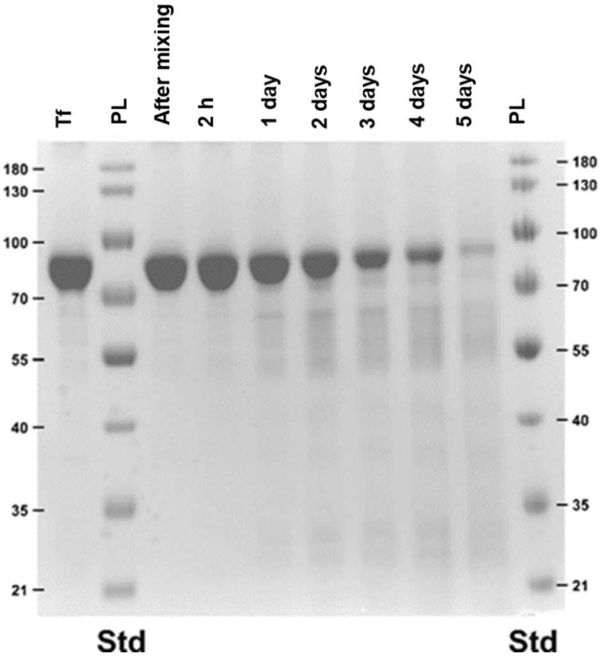

Figure S2. SDS-PAGE gel of transferrin (7.5 μM) in water (lane 1) and transferrin (7.5 μM) incubated in phosphate buffer (10 mM, pH 7.4) with **Zr-WD 4:2** (0.75 mM) at 60 °C for different time increments (lanes 3–9).

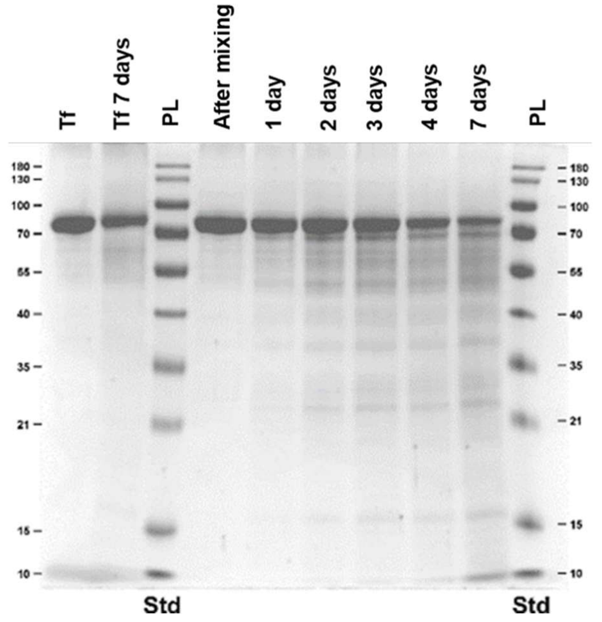

Figure S3. SDS-PAGE gel of transferrin (7.5 μM) in water (lane 1), transferrin (7.5 μM) incubated in phosphate buffer (10 mM, pH 7.4) for 7 days at 60 °C (lane 2) and transferrin (7.5 μM) incubated in phosphate buffer (10 mM, pH 7.4) with **Zr-K 1:2** (0.75 mM) at 60 °C for different time increments (lanes 4–9).

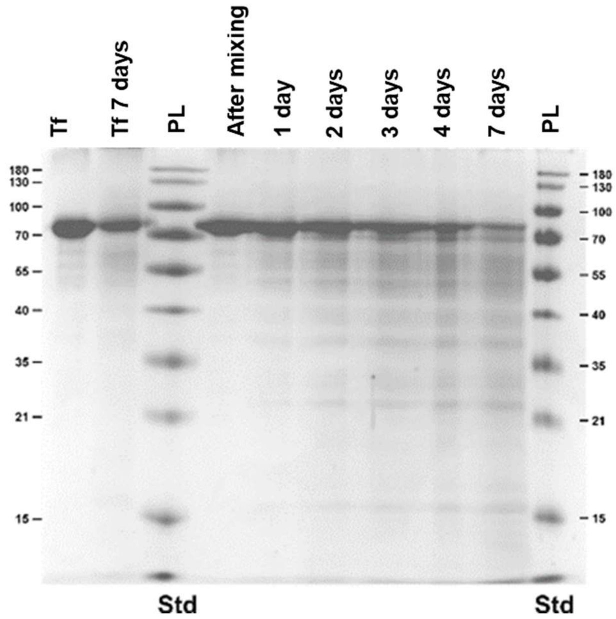

Figure S4. SDS-PAGE gel of transferrin (7.5 μM) in water (lane 1), transferrin (7.5 μM) incubated in phosphate buffer (10 mM, pH 7.4) for 7 days at 60 °C (lane 2) and transferrin (7.5 μM) incubated in phosphate buffer (10 mM, pH 7.4) with **Zr-K 2:2** (0.75 mM) at 60 °C for different time increments (lanes 4–9).

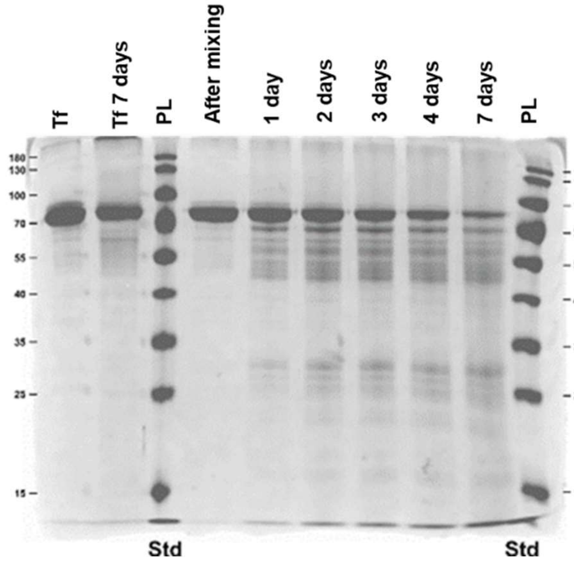

Figure S5. SDS-PAGE gel of transferrin (7.5 μM) in water (lane 1), transferrin (7.5 μM) incubated in phosphate buffer (10 mM, pH 7.4) for 7 days at 60 °C (lane 2) and transferrin (7.5 μM) incubated in phosphate buffer (10 mM, pH 7.4) with **Zr-L 1:1** (0.75 mM) at 60 °C for different time increments (lanes 4–9).

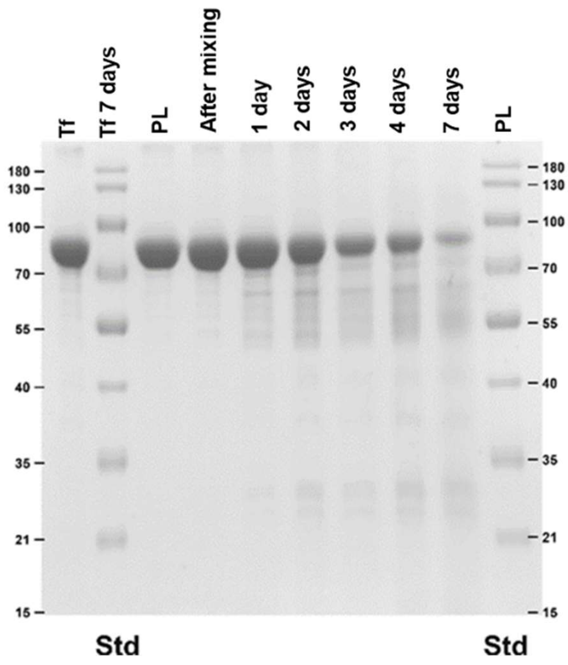

Figure S6. SDS-PAGE gel of transferrin (7.5 μM) in water (lane 1) and transferrin (7.5 μM) incubated in phosphate buffer (10 mM, pH 7.4) with **Zr-L 2:2** (0.75 mM) at 60 °C for different time increments (lanes 3–9).

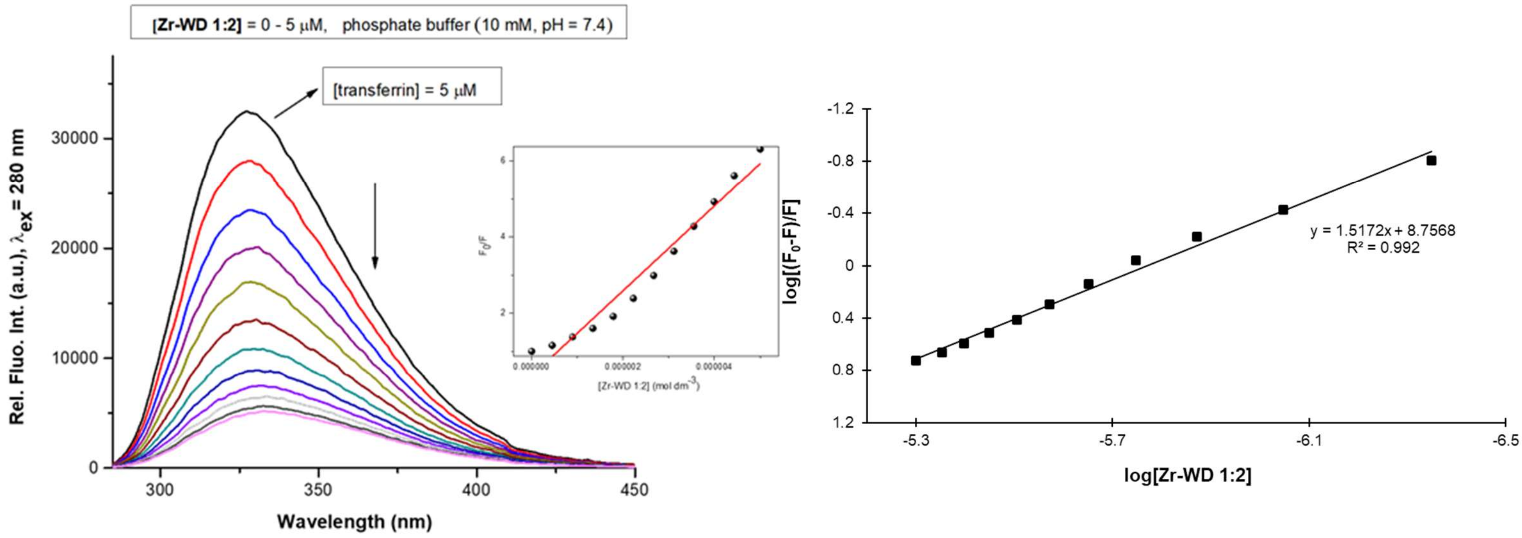

Figure S7. Tryptophan fluorescence quenching spectra of transferrin (5 μM) in phosphate buffer (10 mM, pH 7.4) with increasing concentrations of **Zr-WD 1:2** (0–5 μM). The insert shows a plot of F_0/F versus the POM concentration. (Right) Derived Stern-Volmer plot used to calculate the association constant.

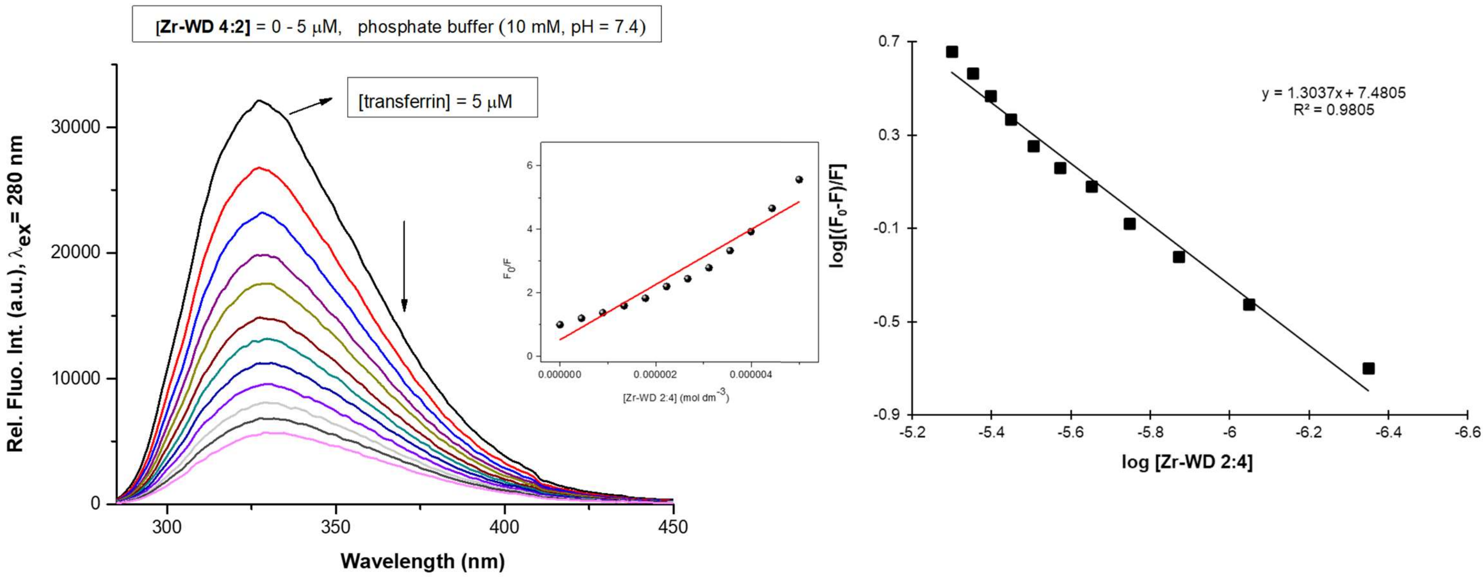

Figure S8. Tryptophan fluorescence quenching spectra of transferrin (5 μM) in phosphate buffer (10 mM, pH 7.4) with increasing concentrations of **Zr-WD 4:2** (0–5 μM). The insert shows a plot of F_0/F versus the POM concentration. (Right) Derived Stern-Volmer plot used to calculate the association constant.

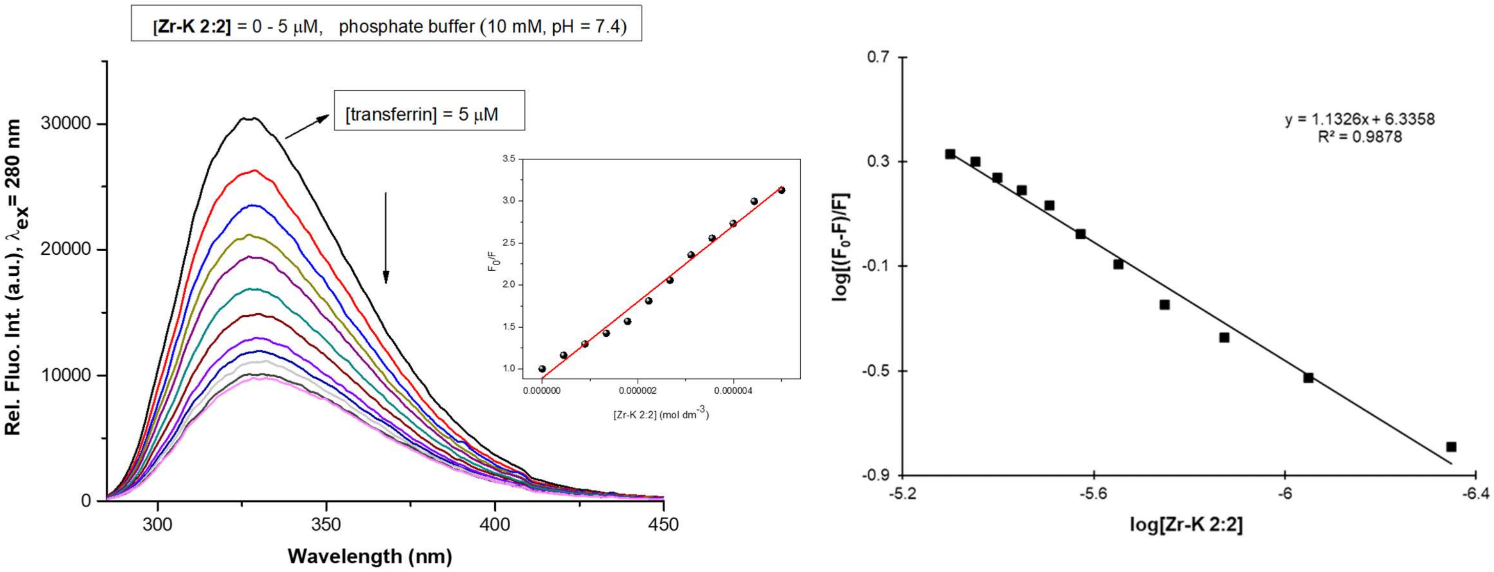

Figure S9. Tryptophan fluorescence quenching spectra of transferrin (5 μM) in phosphate buffer (10 mM, pH 7.4) with increasing concentrations of **Zr-K 2:2** (0–5 μM). The insert shows a plot of F_0/F versus the POM concentration. (Right) Derived Stern-Volmer plot used to calculate the association constant.

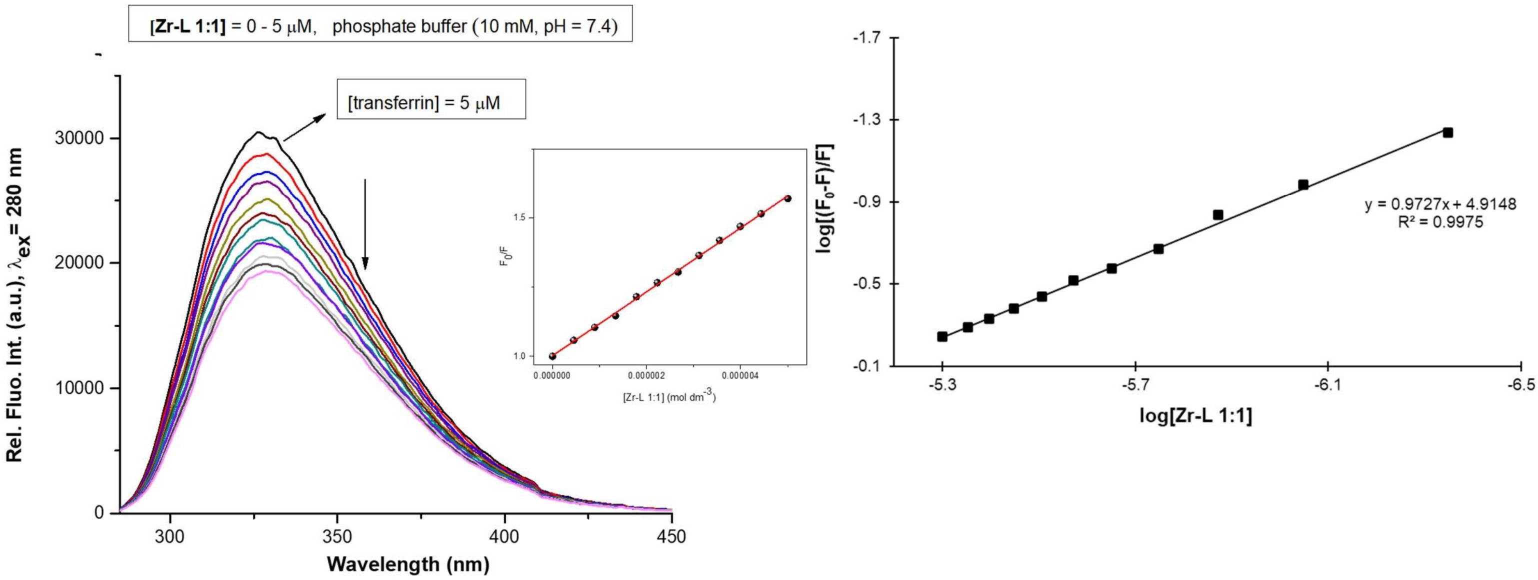

Figure S10. Tryptophan fluorescence quenching spectra of transferrin (5 μM) in phosphate buffer (10 mM, pH 7.4) with increasing concentrations of **Zr-L 1:1** (0–5 μM). The insert shows a plot of F_0/F versus the POM concentration. (Right) Derived Stern-Volmer plot used to calculate the association constant.

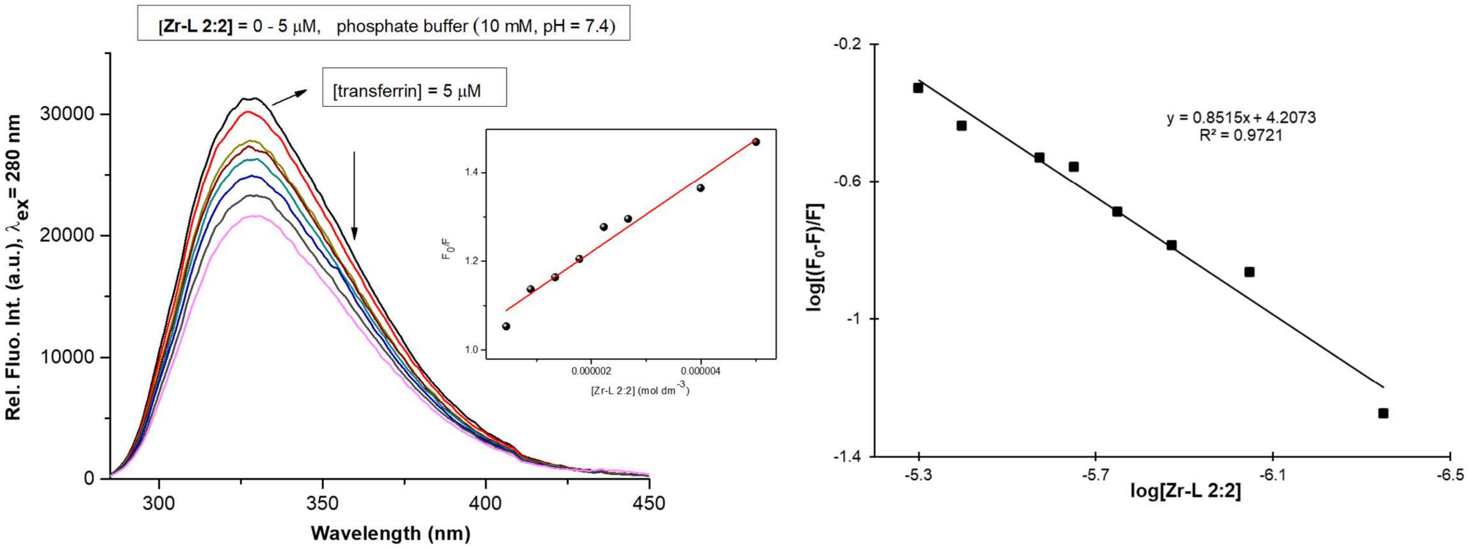

Figure S11. Tryptophan fluorescence quenching spectra of transferrin (5 μM) in phosphate buffer (10 mM, pH 7.4) with increasing concentrations of **Zr-L 2:2** (0–5 μM). The insert shows a plot of F_0/F versus the POM concentration. (Right) Derived Stern-Volmer plot used to calculate the association constant.

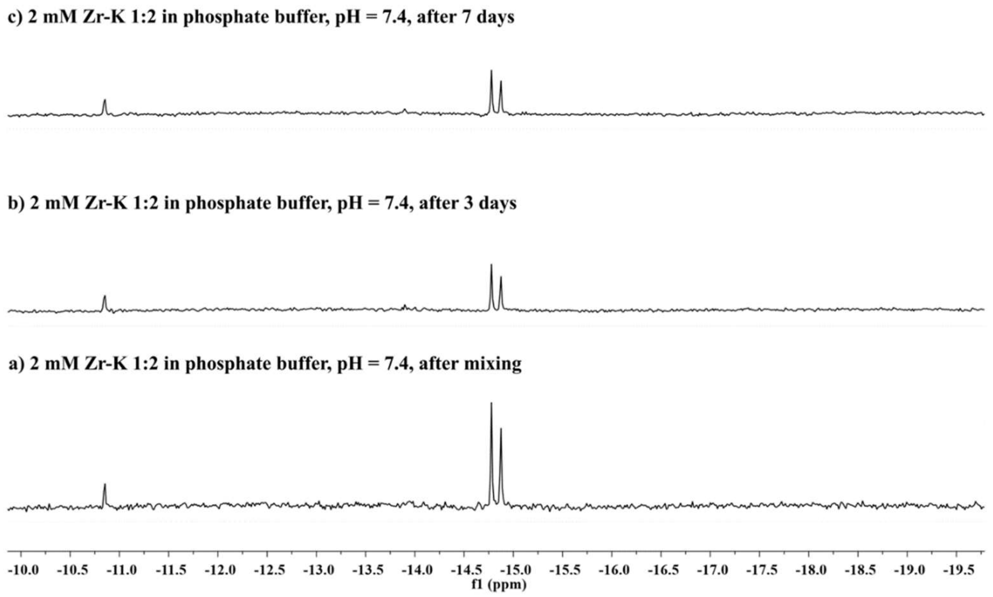

Figure S12. ^{31}P NMR of **Zr-K 1:2** (2 mM) in phosphate buffer (10 mM, pH 7.4) at room temperature. The **Zr-K 1:2** structure (−14.78 and −14.83 ppm) remains largely stable over time, the signal at −10.85 ppm is attributed to the lacunary Keggin.

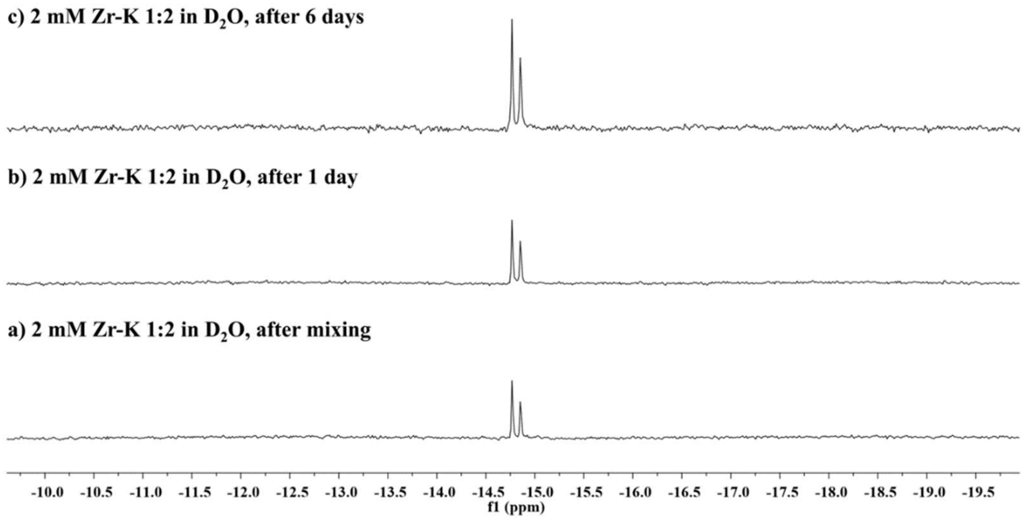

Figure S13. ^{31}P NMR of **Zr-K 1:2** (2 mM) in D_2O at room temperature. The **Zr-K 1:2** structure (−14.78 and −14.83 ppm) remains largely stable over time.

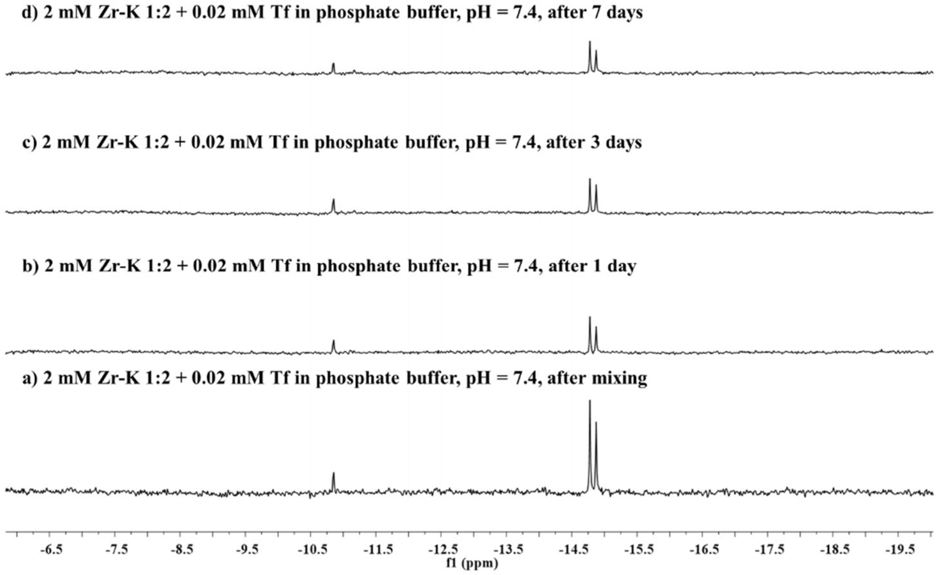

Figure S14. ^{31}P NMR of **Zr-K 1:2** (2 mM) in the presence of transferrin (20 μM) incubated in phosphate buffer (10 mM, pH 7.4) at 60 °C. The **Zr-K 1:2** structure (−14.78 and −14.83 ppm) remains largely stable over time, the signal at −10.85 ppm is attributed to the lacunary Keggin.

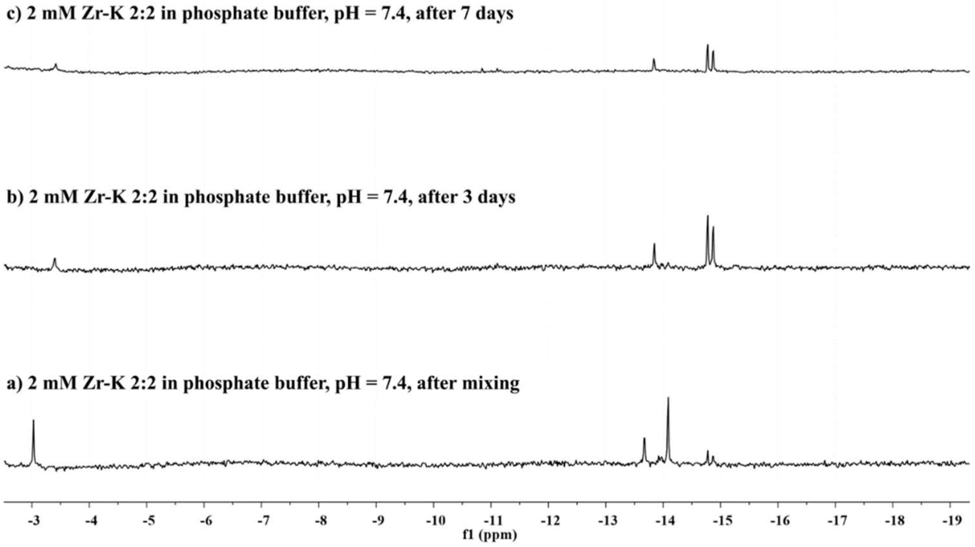

Figure S15. ^{31}P NMR of **Zr-K 2:2** (2 mM) in phosphate buffer (10 mM, pH 7.4) at room temperature. The peak corresponding to intact **Zr-K 2:2** appears at −13.69 ppm in buffer solution. Simultaneously, 2 peaks corresponding to **Zr-K 1:2** appeared around −14.77 and 14.86 ppm. Additional peaks appeared around −14 ppm and one around −3 ppm.

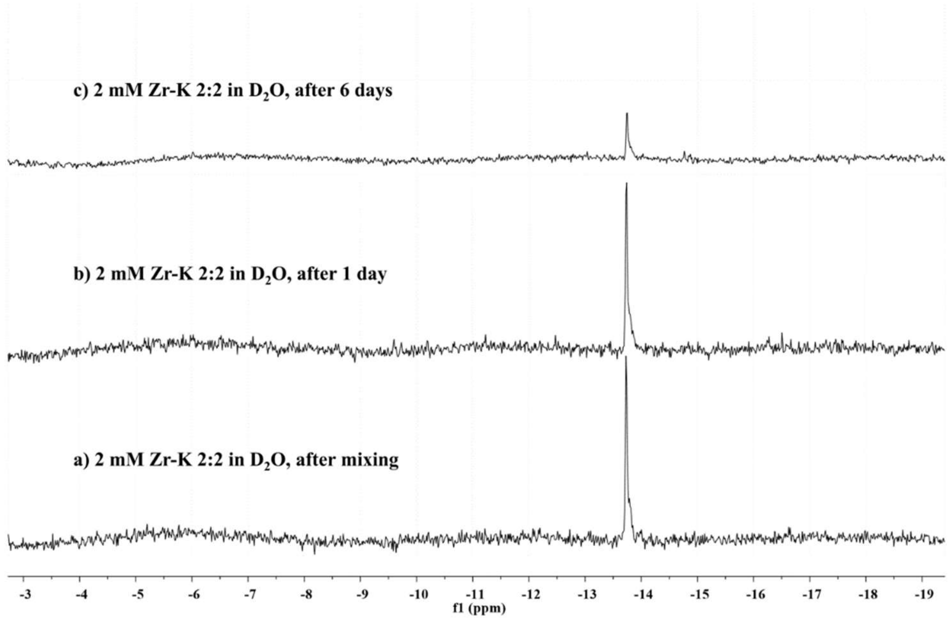

Figure S16. ^{31}P NMR of **Zr-K 2:2** (2 mM) incubated in D_2O at room temperature. The **Zr-K 2:2** structure (−14.78 and −14.83 ppm) remains largely stable over time.

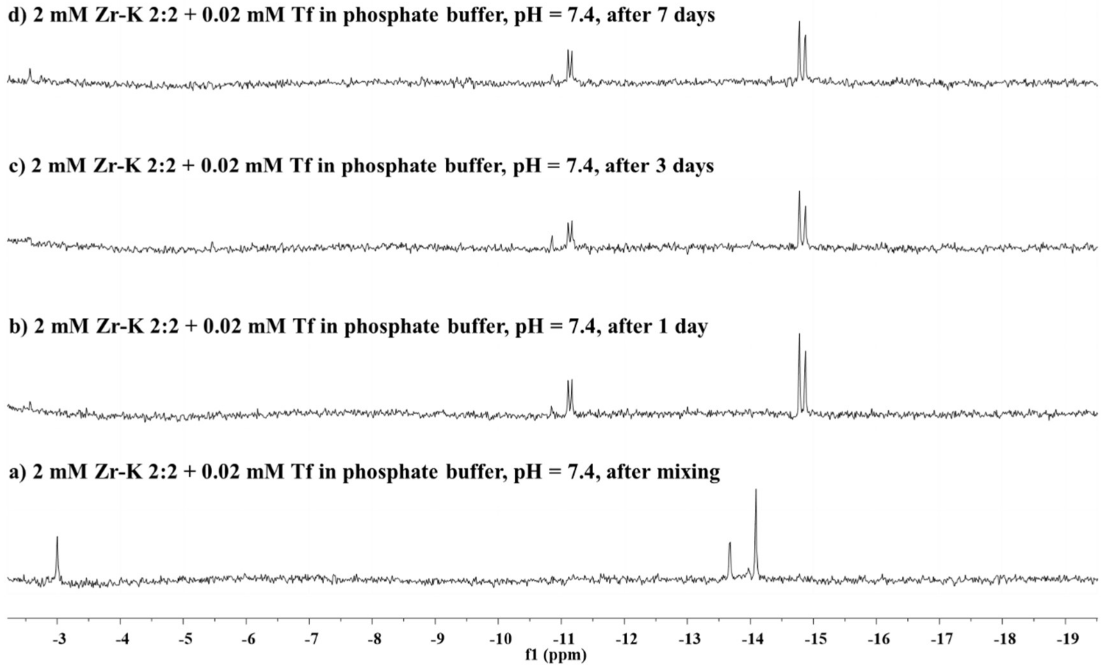

Figure S17. ^{31}P NMR of **Zr-K 2:2** (2 mM) incubated in phosphate buffer (10 mM, pH 7.4) at 60 °C in the presence of transferrin (20 μM). The peak corresponding to intact **Zr-K 2:2** appears at −13.69 ppm in buffer solution, this peak disappeared after 1 day of incubation. Simultaneously, 2 peaks corresponding to **Zr-K 1:2** appeared around −14.77 and 14.86 ppm. Additional peaks appeared around −14 ppm and one around −3 ppm immediately after mixing. In all conditions, **Zr-K 2:2** was quickly converted to **Zr-K 1-2** upon incubation. Other unknown peaks appeared around −11 ppm.

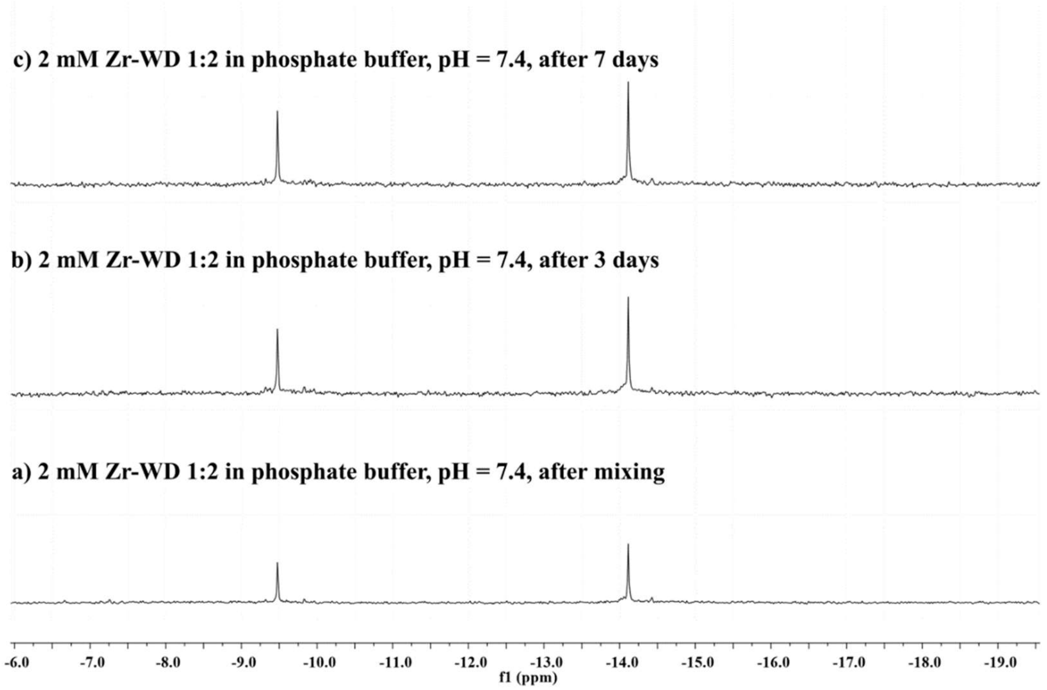

Figure S18. ^{31}P NMR of **Zr-WD 1:2** (2 mM) in phosphate buffer (10 mM, pH 7.4) at room temperature. The **Zr-WD 1:2** structure (−14.11 and −9.48 ppm) remains largely stable over time.

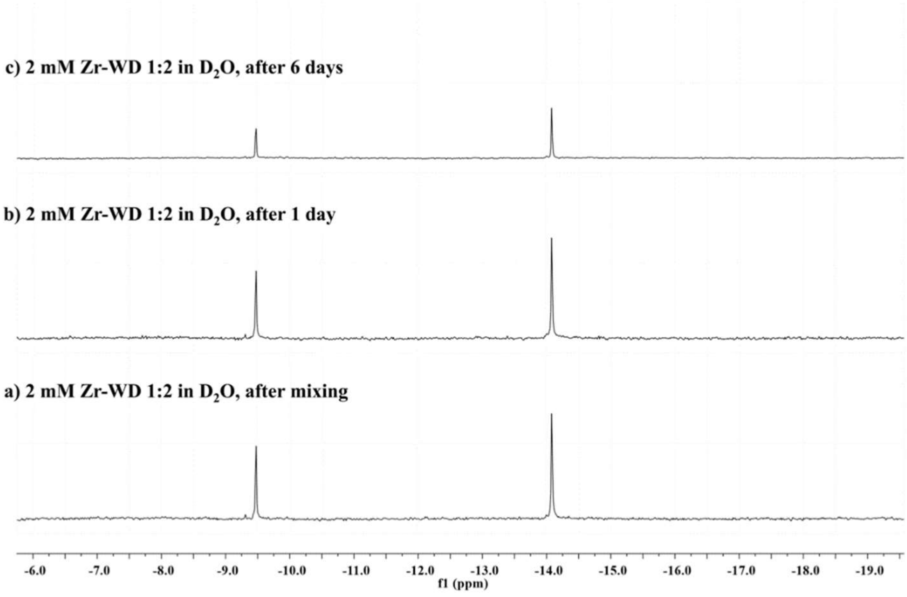

Figure S19. ^{31}P NMR of **Zr-WD 1:2** (2 mM) in D_2O at room temperature. The **Zr-WD 1:2** structure (−14.08 and −9.47 ppm) remains largely stable over time.

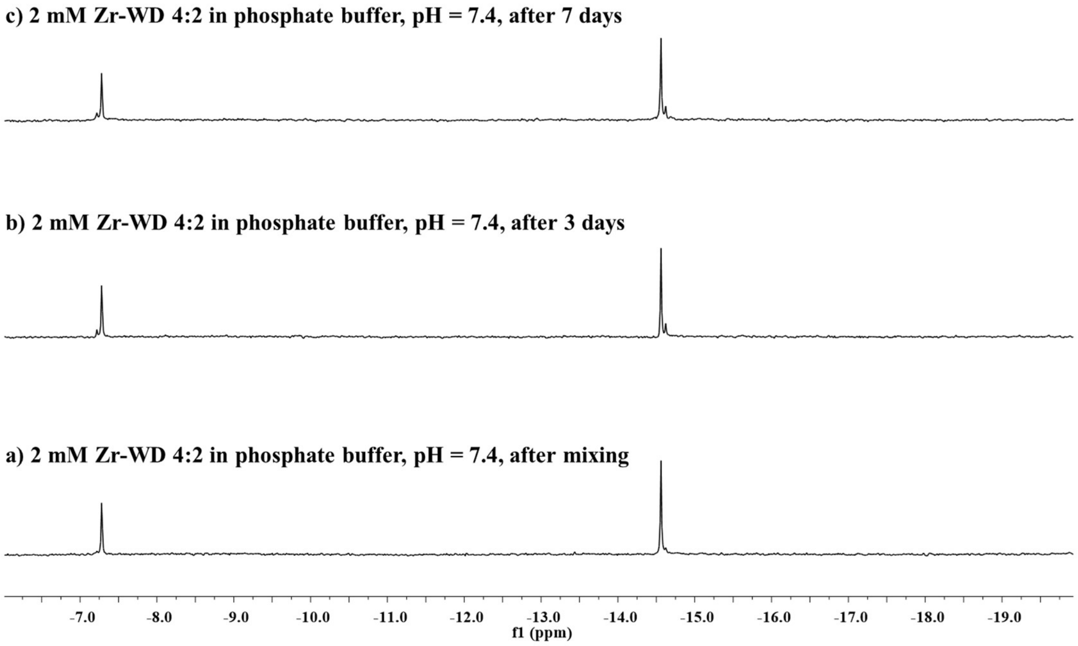

Figure S20. ^{31}P NMR of **Zr-WD 4:2** (2 mM) in phosphate buffer (10 mM, pH 7.4) at room temperature. The **Zr-WD 4:2** structure (−14.59 and −7.28 ppm) remains largely stable over time.

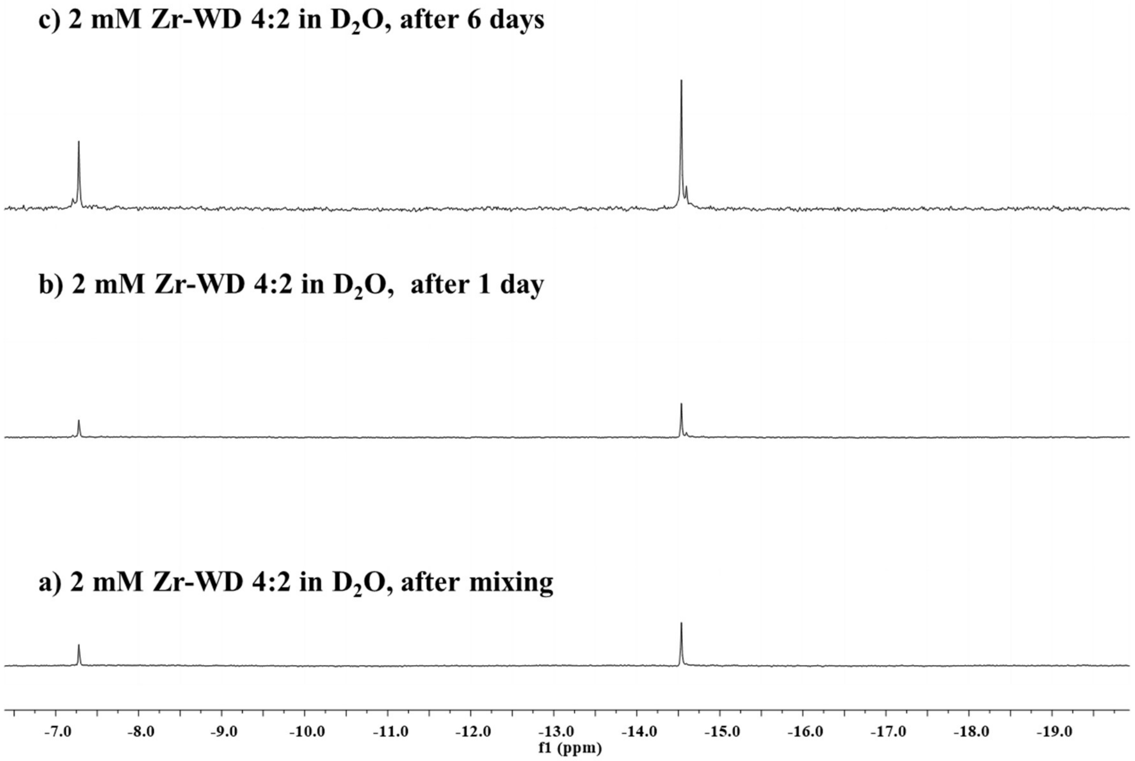

Figure S21. ^{31}P NMR of **Zr-WD 4:2** (2 mM) in D_2O at room temperature. The **Zr-WD 4:2** structure (−14.59 and −7.28 ppm) remains largely stable over time.

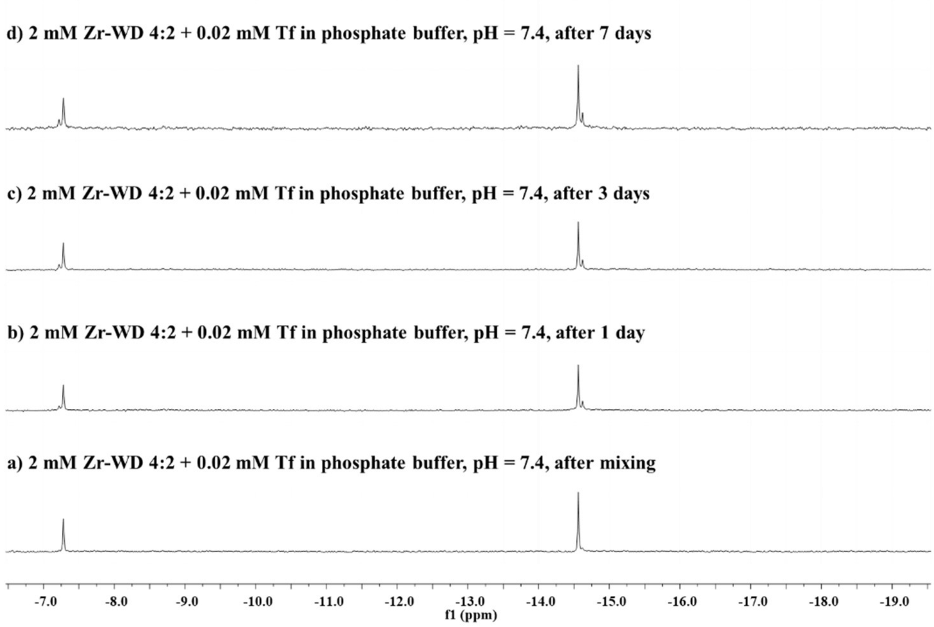

Figure S22. ^{31}P NMR of **Zr-WD 4:2** (2 mM) incubated in phosphate buffer (10 mM, pH 7.4) at 60 °C in the presence of transferrin (20 μM). The **Zr-WD 4:2** structure (−14.59 and −7.28 ppm) remains largely stable over time.

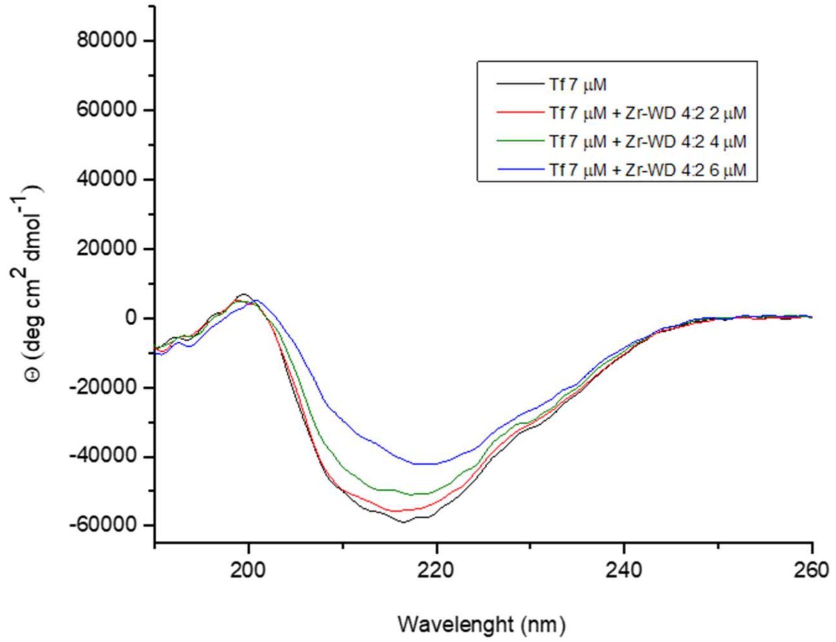

Figure S23. CD spectra of transferrin (7 μM) in phosphate buffer (10 mM, pH 7.4) with increasing concentrations (0–10 μM) of **Zr-WD 4:2**.

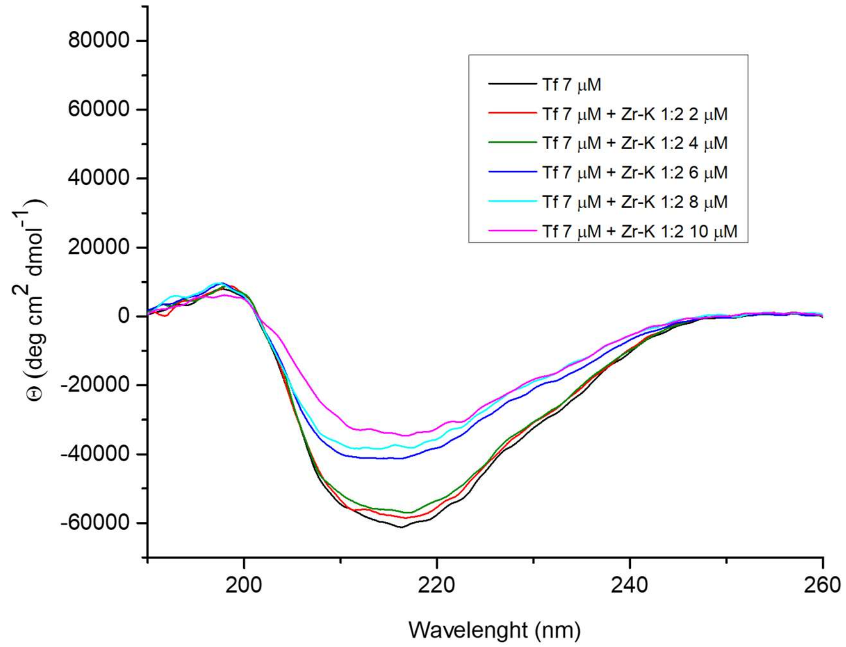

Figure S24. CD spectra of transferrin (7 μM) in phosphate buffer (10 mM, pH 7.4) with increasing concentrations (0–10 μM) of **Zr-K 1:2**.

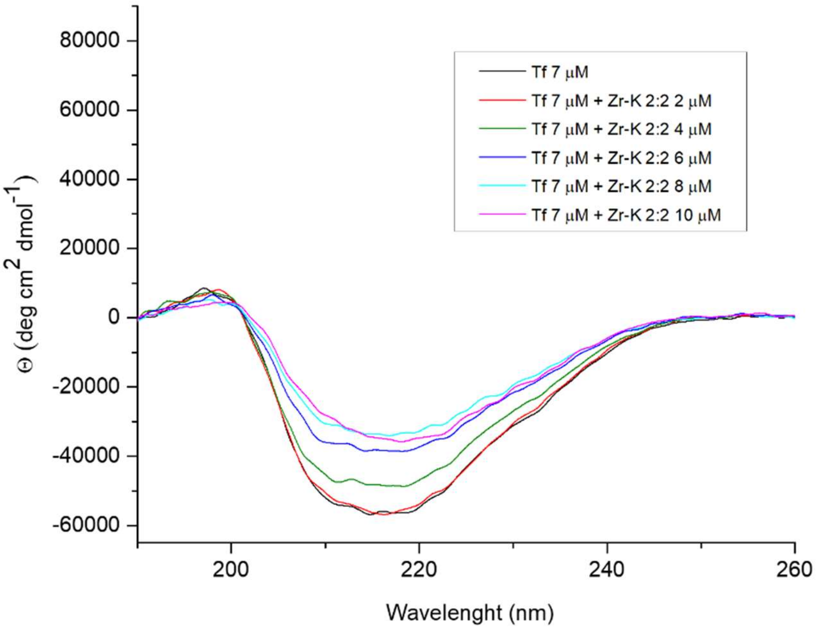

Figure S25. CD spectra of transferrin (7 μM) in phosphate buffer (10 mM, pH 7.4) with increasing concentrations (0–10 μM) of **Zr-K 2:2**.

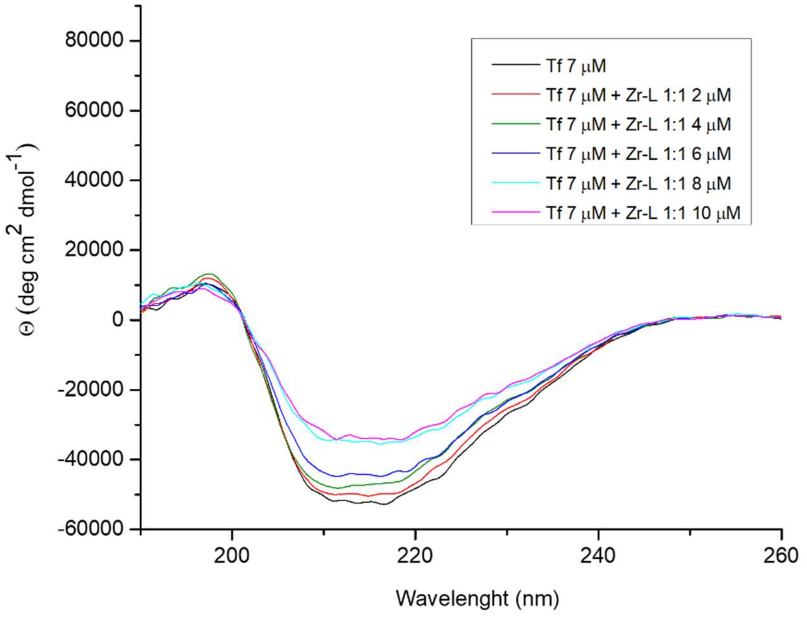

Figure S26. CD spectra of transferrin (7 μM) in phosphate buffer (10 mM, pH 7.4) with increasing concentrations (0–10 μM) of **Zr-L 1:1**.

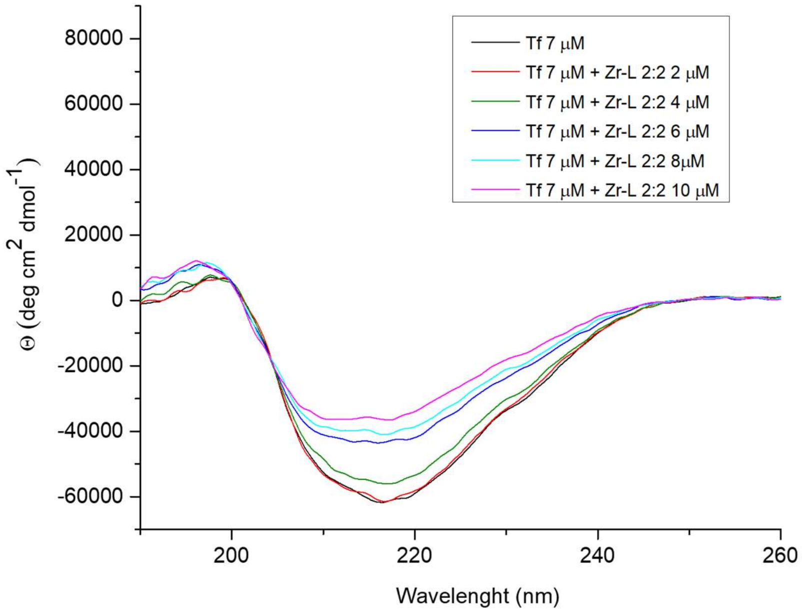

Figure S27. CD spectra of transferrin (7 μM) in phosphate buffer (10 mM, pH 7.4) with increasing concentrations (0–10 μM) of **Zr-L 2:2**.
